# Neural Tube Defects in Resource‐Limited Settings: A Lebanese Case Series Highlighting Prevention Gaps and Challenges in Prenatal Diagnosis

**DOI:** 10.1155/crog/2044243

**Published:** 2026-09-30

**Authors:** Georges Yared, Charlotte El Hajjar, Nidal Hammoud, Christopher Massaad, Kariman Ghazal

**Affiliations:** ^1^ Obstetrics and Gynecology Department Lebanese American University Beirut Lebanon lau.edu.lb; ^2^ Department of Obstetrics and Gynecology Faculty of Medical Sciences Lebanese University Beirut Lebanon ul.edu.lb; ^3^ Faculty of Medicine Saint George University of Beirut Beirut Lebanon

## Abstract

**Background:**

Neural tube defects (NTDs) are congenital developmental anomalies resulting from incomplete neural tube closure during early embryonic development. They present a wide range of clinical manifestations along the craniospinal axis.

**Objective:**

With only 6.2% of Lebanese women of childbearing age taking folic acid tablets during the adequate period, it is primordial to shed light on cases of NTDs in Lebanon. This case series presents four cases of NTDs that deviate from the conventional “zipper closure” model of neural tube development, aligning more with the modern multisite closure approach. This case series showcases the consequences of inadequate intake of folic acid prior to conception in addition to delayed prenatal diagnosis. The four cases are presented in accordance with CARE reporting guidelines.

**Methods:**

The study presents four case reports of NTDs, including iniencephaly, anencephaly, encephalocele, and spina bifida. Each case details the clinical presentation, diagnostic findings, and outcomes.

**Results:**

The cases demonstrate various manifestations of NTDs: (1) a case of iniencephaly with hydrocephalus and spina bifida in a 32‐week fetus, (2) a case of anencephaly in a 34‐week fetus, (3) a case of encephalocele in one fetus of diamniotic dichorionic twins at 37 weeks, and (4) a case of closed spina bifida diagnosed at 36 weeks.

**Discussion:**

The article explores the embryology, classification, and epidemiology of NTDs. It discusses factors that can contribute to NTDs. We also note the limited prenatal follow‐up and care in vulnerable populations in Lebanon. Genetic and environmental factors are also discussed, with a particular focus on the role of folic acid and its metabolism.

**Conclusion:**

This case series highlights the diverse presentations of NTDs. It further showcases the importance of early diagnosis and management. It also emphasizes the need for folic acid supplementation and the consideration of multiple etiologies in the prevention and understanding of NTDs.

## 1. Introduction

Neural tube defects (NTDs) are congenital developmental anomalies. They result from incomplete neural tube closure during the early embryonic stage. This primary defect is most often associated with secondary abnormalities. They mainly occur in the formation of surrounding mesodermal structures. These include bony and muscular structures that typically englobe neural tissues. The resulting anomalies can manifest along the craniospinal axis. They can also have different clinical presentations. Because of this high diversity, advanced imaging remains crucial for diagnosis and management. In particular, it is rare to see NTDs at different locations along the craniospinal axis. Indeed, they account for less than 1% of all documented NTD cases [1, 2].

On a global level, NTDs affect approximately 1 in 1000 live births. Its rates are disproportionate in low to middle income countries (LMICs). Indeed, these populations tend to have more nutritional deficiencies and more limited access to antenatal care [3] The estimated average global prevalence is two cases per 1000 births. On annual basis, it amounts to approximately 214,000 to 322,000 pregnancies worldwide [4].

In Lebanon, data on the incidence of NTD incidence remains scarce. Indeed, in 2021, Lebanon had an age standardized incidence of 1.17/100,000. This number is consistent with neighboring countries. This includes Jordan (0.77 per 100,000) and Syria (1.20 per 100,000) [5]. However, these numbers might be underestimations. This is mainly because of the absence of a mandatory national registry for congenital anomalies in Lebanon.

On another level, late diagnosis of NTDs can occur. It may be because of systemic deficiencies in prenatal care, especially among migrants or refugee workers. In addition, the economic crisis in Lebanon could delay antenatal follow‐up [6]. Indeed, according to Chebl et al. (2025), prenatal ultrasound screening remains inconsistently applied in Lebanon [7].

In our clinical experience, we encountered four cases that deviated from the conventional, classical “zipper closure” model of neural tube development [2]. After a literature review on NTD embryogenesis, we found that these cases are more consistent with the modern multisite closure. This theory provides a more comprehensive framework in order to understand these anomalies.

This manuscript was written in adherence with the CARE Case Report guidelines [8].

Indeed, the primary objective is to document the clinical presentation, findings, and outcomes of the four NTD cases. We further attempt to situate these cases within the broader literature, especially in Lebanon while advocating for targeted antenatal health interventions. A secondary aim of this study is to discuss the embryological basis of these defects especially when it comes to the theory of multisite neural tube closure.

## 2. Case Report 1

A 17‐year‐old primigravida woman presented to the Obstetrics and Gynecology Department at Rafik Hariri University Hospital for active labor and severe pain. She had a nonconsanguineous marriage. She had not taken any iron or folic acid supplements. She reported that her routine blood tests and biochemical parameters were within normal ranges.

Uterine size was consistent with 32 weeks of gestation. The patient presented with rupture of membranes and labor. Ultrasound showed a live fetus with hydrocephalus and iniencephaly. The biparietal diameter surpassed 100 mm.

The patient underwent a spontaneous vaginal delivery. A female neonate weighing 1350 g was born with severe congenital anomalies. The newborn displayed settled retroflection of the head, nonappearance of the neck, low‐set ears, and a lumbar meningocele. Physical examination of the neonate revealed significant craniofacial and spinal abnormalities. The head was markedly enlarged and severely retroflexed with no discernible neck. The mandibular skin appeared continuous with the chest surface. Low‐set ears were observed, as shown in (Figure 1A). The vertebral column was notably short and irregular. The lower limbs appeared normal. However, the upper limbs showed disproportionate growth compared with the lower extremities (Figure 1B).

**Figure 1:**
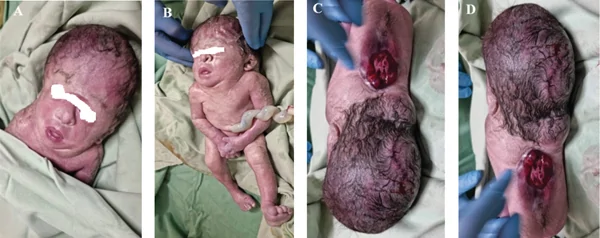
Clinical photographs of the newborn with iniencephaly, hydrocephalus, and spina bifida. (A) Marked enlargement and severe retroflexion of the head, absence of a discernible neck, and low‐set ears. (B) Disproportionate appearance of the upper limbs compared with the lower extremities. (C) Posterior view showing extreme hyperextension of the neck and a large open spinal defect consistent with spina bifida. (D) Posterior craniospinal abnormalities with thoracolumbar lordosis, supporting the diagnosis of iniencephaly.

Posterior examination revealed thickened scalp skin extending to the lumbar region. There was extreme hyperextension of the neck, with the occiput in contact with the cervicodorsal vertebrae and continuous soft tissue on the dorsal aspect of the neck. A large, high open spina bifida was present, accompanied by posterior cervical and thoracic vertebral anomalies (Figure 1C). Defective block vertebrae and thoracolumbar lordosis were observed, further supporting the diagnosis of iniencephaly (Figure 1D). Appallingly, she surrendered 10 min after birth. Because of the family′s refusal of postmortem examination and karyotype, a comprehensive assessment for extra anomalies was not conceivable.

## 3. Case Report 2

A 22‐year‐old primigravida presented to the prenatal care of Obstetrics and Gynecology Department of Rafik Hariri University Hospital for her to begin with a prebirth visit. She had a history of first‐degree consanguineous marriage and had not taken any iron or folic acid supplements. Her scheduled blood tests and biochemical parameters were within ordinary limits. Abdominal examination uncovered a uterus reliable with 34 weeks of gestation. The patient was admitted with ruptured membranes and contractions. Ultrasonography uncovered a live baby with anencephaly. The mother delivered a 1250‐gram female fetus with anencephaly (Figure 2), which passed on 10 min after birth.

**Figure 2:**
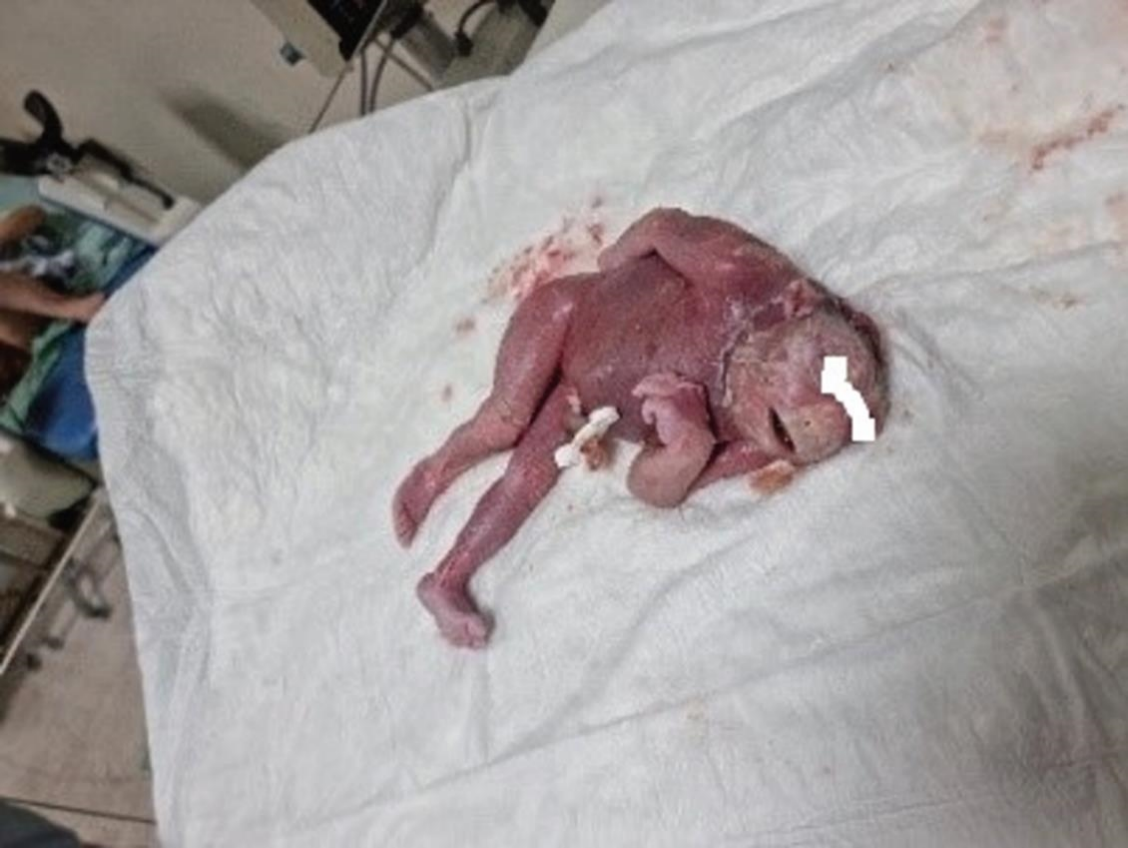
Anencephalic newborn.

As the family declined a postmortem examination and karyotype, a comprehensive assessment for extra anomalies was not conceivable.

## 4. Case Report 3

A 27‐year‐old woman, Gravida 3, Para 2 with, one past cesarean section and one abortion at 5 weeks, presented to the obstetrics clinic for her initial prenatal visit. She detailed a nonconsanguineous marriage and had not been taking iron or folic acid supplements. Her scheduled blood tests and biochemical parameters were within ordinary limits.

The primary ultrasound scan, performed at 19 weeks and 5 days gestation, uncovered diamniotic dichorionic twins. Fetus A displayed an unusual brain encephalocele, whereas Fetus B appeared normal.

2D and 3D ultrasounds were done. The sonogram affirmed a viable Fetus A, weighing 405 g at 19 weeks and 3 days gestation. A large, high occipital posterior bony defect was observed, featuring a protruding mass characteristic of an encephalocele, measuring 4.5 cm in its largest dimension. The cerebellum appeared hypoplastic. It measured 1.55 cm and was contained within the defect along with the lateral ventricles and choroid plexus. Head measurements were below average for gestational age. They indicated microcephaly. All other anatomical measurements were within normal limits. The patient declined amniocentesis. Follow‐up growth assessment, biophysical profile (BPP), and a 2D sonogram were performed at 33 weeks and 3 days of gestation. They revealed findings similar to the initial anatomical examination. The BPP score was 8/8. Estimated fetal weight (EFW) was below the second percentile for gestational age, at approximately 1000 g.

The woman underwent monitoring twice per week. These included nonstress tests and BPPs. Discussions were organized with pediatric neurosurgery and neurology departments. At 37 weeks, a cesarean section was performed, resulting in the birth of a female in good condition, with an Apgar score of 9, and a male with high occipital posterior encephalocele (Figure 3A–C), with an Apgar score of 9. Be that as it may, the newborn experienced respiratory distress and was transferred to the NICU. Tragically, he subsequently succumbed to sepsis under the supervision of the neonatological team.

**Figure 3:**
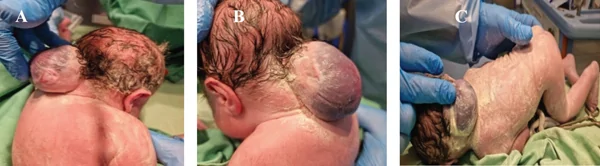
Clinical photographs of the male newborn with a high occipital posterior encephalocele. (A–C) Different clinical views demonstrating the large posterior occipital encephalocele.

## 5. Case Report 4

A 30‐year‐old lady, Gravida 5, Para 3 (G5P3A1), presented for a routine anatomical sonogram in the Obstetrics and Gynecology Department at Rafik Hariri University Hospital at 33 weeks and 3 days of development, based on her last menstrual period. This was her initial ultrasound examination for the current pregnancy. The patient had not been taking prebirth vitamins or folic acid supplements.

The patient′s family history was negative for any NTDs. A transabdominal ultrasound was performed with a high resolution transducer with 3D imaging. The examination revealed a midline deformity within the lumbosacral region of the fetal spine. Advance evaluation showed a sac‐like protrusion containing CSF, reliable with closed spina bifida.

Following the diagnosis, the woman was counseled by pediatric neurosurgery and neurology specialists. At 37 weeks and 5 days gestation, she was admitted for labor. A pelvic examination uncovered total cervical dilation and breech presentation. An emergency cesarean section was performed, resulting in the delivery of a male newborn child with an Apgar score of 8. He was instantly transferred to the nursery under the pediatric care. The patient was discharged 2 days afterward with instructions to follow up with a neurosurgeon (Figure 4).

**Figure 4:**
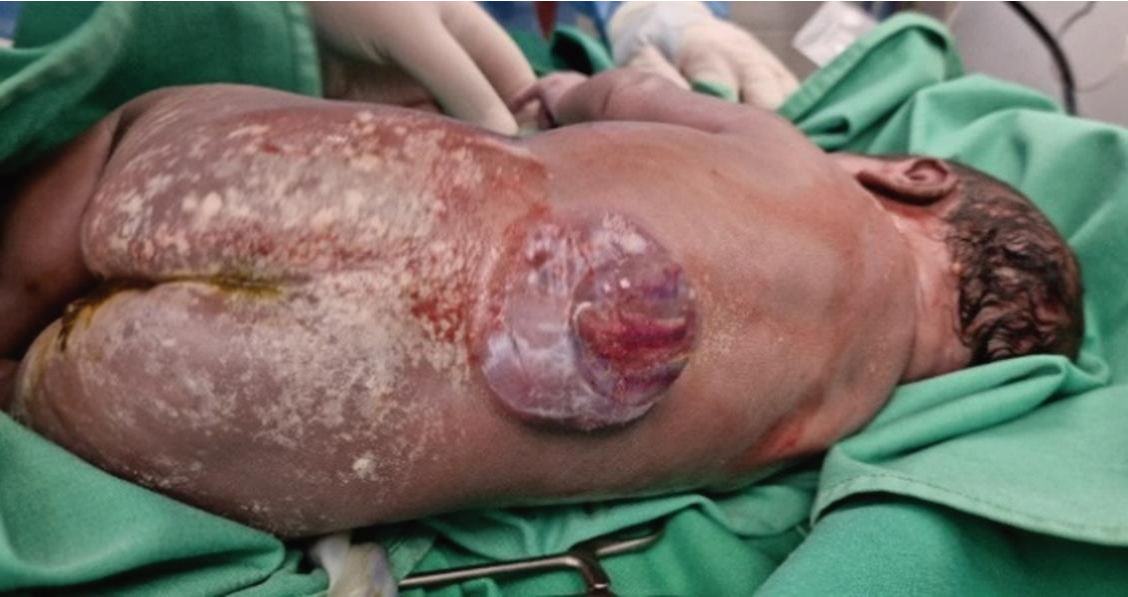
Picture of the newborn with spina bifida.

## 6. Discussion

NTDs are a common, severe central nervous system defect that occurs early in pregnancy. As early as the third week, a complex morphogenic process called neurulation begins, involving the closure of anterior and posterior neuropores through neurulation. This process is influenced by the notochord and prechordal mesoderm. Neurulation occurs in two stages: primary and secondary. Primary neurulation involves folding the dorsal neural plate to construct the brain and upper spinal cord, whereas secondary neurulation involves canalizing neural tubes to create the lower back portion of the spine [1].

The four cases in this series represent failures of prevention. They also showcase the structural deficiencies of prenatal care in Lebanon. Although they do not serve as direct evidence for the embryological closure theory, the topographic pattern of defects (cranial, cervicothoracic, occipital, and lumbosacral) is more consistent with the multisite closure model rather than the classical zipper hypothesis.

Numerous molecular variables have also been found to affect this process; the majority of them are related to the start of neural plate development and include bone morphogenetic Protein 4 (BMP4) suppression and fibroblast growth factor (FGF) signaling. Additionally, by controlling the expression of the homeobox gene, retinoic acid plays a crucial role in establishing the shape of the cranial–caudal axis [2]. From the brain to the sacrum, NTDs can appear anywhere along the neuroaxis. They fall into two general categories: open faults and closed flaws. Primary neurulation failure causes open NTDs, which can result in exposed neural tissue and cerebrospinal fluid leaks, as well as consequences, such as hydrocephalus and Chiari II malformation. Secondary neurulation failure causes closed NTDs, which are usually limited to the spinal cord and can manifest as lipomas that have dorsal abnormalities, such lipomyelocele or lipomyelomeningocele.

Several separate situations are included in the categorization of NTDs which are further depicted in Table 1 and Figure 5 [9].

**Table 1:** Categorization of neural tube defects.

Neural tube defect	Characteristics
Anencephaly	Complete or partial absence of the brain, skull, and surrounding skin is known as cranial vault absence.
Craniorachischisis	Extensive neural tube opening.
Iniencephaly	A combination of spinal defect and cranial vault absence with exposed neural tissue and occipital dysraphism. This rare disorder causes the head and trunk to tilt severely backward.
Spinal dysraphism (spina bifida)	Protrusion of neural tissue and meninges through a spinal bone defect that may be open or covered in skin. It can occur at any level of the spine.
Encephalocele	An extension of brain tissue and meninges through a defect in the skull, which can occur in the frontal, nasofrontal, occipital, parietal, orbital, or nasal areas, is known as cranial meningocele.

**Figure 5:**
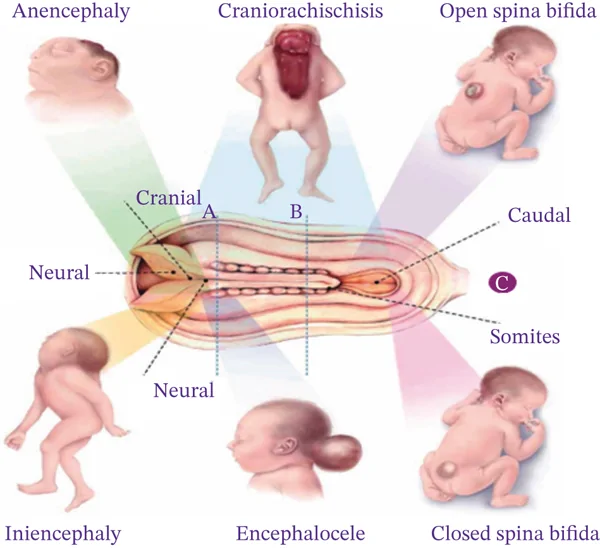
Schematic illustration of neural tube defects resulting from abnormal neural tube closure. A–C indicate the labeled neural tube closure regions shown in the schematic. Failure of closure at different levels may result in anencephaly, craniorachischisis, open spina bifida, iniencephaly, encephalocele, or closed spinal dysraphism.

Several risk determinants of NTDs, such as immune system defects, obesity, diabetes, folate antagonism, socioeconomic status, and geographic and ethnic factors, need to be gathered together and confirmed by further investigations. There is also a geographic and ethnic component to these abnormalities. Occupational forces of low socioeconomic status cause the shortfall of folic acid along with prenatal nutrition. Maternal fever, nitrosating agents, and amniotic bands may also contribute in a hindering way to the development of the neural tube [10, 11]. To a certain degree, the physical and chemical environment also plays a role, including elements like radiation, stress, Hypervitaminosis A, rubella, toxoplasmosis, cytomegalovirus, and hazardous waste from landfills within 3 km [12].

Since the year 1980, the Centers for Disease Control recommended folic acid on a daily basis for every pregnant woman owing to a 71% drop in noncommunicable disease (NCDs) among babies born to women who utilized multivitamins early in pregnancy or even before it [3, 13]. Folate, important for the de novo synthesis of DNA and RNA precursors, is absolutely crucial as a methyl donor during 5‐methyltetrahydrofolate (5‐MTHF) production [14, 15].

A large population of women does have enough quantities of folic acid during pregnancy and do give birth, yet their infants exhibit congenital spinal or cranial defects called NTDs. This is particularly relevant in the Lebanese context, where these four case studies were reported. Indeed, with limited statistical data pertaining to NTDs in Lebanon, it remains crucial to shed light on prenatal screening measures while increasing access to prenatal care or folic acid supplementation programs. According to a study conducted by Nasr Hage et al. (2012), only 6.2% of Lebanese women of childbearing age had taken folic acid tablets during the adequate period [16]. This alarming statistic highlights the importance of reporting cases of NTDs, especially in the Lebanese context where limited data is available. Moreover, a subsequent study conducted by Hage and Abi Rizk converged on this limited intake of folic acid and further highlights that although 60% of women knew about folic acid, only 7.5% were using supplements adequately [17].

NTDs can be genetically caused by polymorphic variations in the enzymes synthesizing folate metabolism pathways, folate receptors, or even folate transport proteins. These neuroectodermal cell surface receptor abnormalities are caused by the fetal developmental time course. These membrane protein abnormalities are critical for central nervous system development as they are important for the intake of folate and its alpha and beta derivatives needed during the process of neural tube formation [18]. Temporal expression of neural folds is accompanied by the expression of folate receptors. The MTHFR C677T polymorphism is linked to NTDs, according to meta‐analyses, with research demonstrating that it lowers the activity of enzymes that metabolize folate, resulting in low blood folate levels [19]. Because they counteract the function of folate, the usage of folate antagonists including phenytoin, valproic acid, and carbamazepine directly affects the development of NTDs [20].

One mechanism contributing to NTDs is the inhibition of dihydrofolate reductase by methotrexate, which increases neural tissue death [19]. Furthermore, FGF malfunction and homeobox gene alterations are also associated with NTD pathogenesis. In our cases, the patients did not receive adequate prenatal care and failed to take any supplements before and during pregnancy.

Vitamin B12 is essential for the conversion of folate byproduct homocysteine into methionine through methionine synthase thereby, facilitating the elimination of homocysteine. However, vitamin B12 deficiency leads to the elevation of serum levels of homocysteine. One theory proposes that an elevated concentration of homocysteine causes modified folate receptors through posttranslational modification, leading to homocysteinylation. Because of this process, autoantibodies to these antigens are produced resulting in the reduction of folate activity [15].

Another aspect which should be mentioned is hydrocephalus, which can be responsible for the fetal head circumference to be unable to grow. There is also a common ultrasound examination performed prenatally for the detection of NTDs, which includes finding features such as retardation of the fetal′s head growth, as well as fatigue, pale features in the mother, and anemia [21, 22]. Alpha‐fetoprotein is a globulin protein that is present in the yolk sac, liver, and intestine of the fetus, and it is used in the diagnosis of NTDs. However, this protein does not last long because it is subject to obliteration by the placenta as the pregnancy increases in duration [23] Magnetic resonance imaging (MRI) is used when ultrasound results are inconclusive, but it may interfere with the ultrasound′s functionality [24]. Chromosomal microarray is used to assess the likelihood of carrying genetic mutations related to NTDs [19]. NTDs are often associated with other anomalies, such as congenital cardiac defects, cleft palate, urinary tract, and colon malformations. Maternal serum folic acid levels can also help identify potential causative factors [25, 26].

Our first case of iniencephaly with hydrocephalus and spina bifida is a rare yet invariably fatal NTD. This case highlights the difficulty in diagnosing and the prognostic challenges in resource‐limited settings like Lebanon. Indeed, characteristics such as retroflexion of the head, absence of a neck, and severe vertebral anomalies are consistent with previously reported phenotypes [27] However, the detailed imaging findings, including biparietal diameter surpassing 100 mm and associated hydrocephalus, enrich the literature by providing specific biometric data.

In addition, our second case of anencephaly is one of the most common NTDs. The latter is characterized by the absence of the cranial vault and cerebral hemispheres [28].

Furthermore, this particular case is associated with consanguinity which is still a prevalent practice in Lebanon. Indeed, according to research conducted by Barbour and Salameh (2009), among 1556 women involved in the study, the prevalence of consanguineous marriages was 35.5% [29]. This risk factor conjugated to a lack of folic acid supplementation further puts the fetus at a higher risk of developing NTDs. The rapid neonatal demise highlights how lethal this condition can be. It also pushes toward health education and genetic counseling. It also shows an urgent need for fortification of folic acid programs in Lebanon. Moreover, this case also sheds light on the importance of early prenatal diagnosis, which remains underutilized in Lebanese healthcare settings. The importance of public health interventions is also echoed in our fourth case report. Indeed, although this case of closed spina bifida demonstrates a less severe form of NTD, the latter is still associated with significant morbidity. In this case, the absence of consanguinity and the lack of prenatal vitamin resulted in a modifiable outcome that could have been avoided with adequate prenatal care. For instance, myelomeningocele, the most severe form of spina bifida, can be treated through fetal and postnatal surgery. Postnatal surgery should be done 72 h after birth, with prenatal treatment improving cognitive perception. A ventriculoperitoneal shunt is used for hydrocephalus [30, 31]. Antipyretics can lower NTD chances in hyperthermia. Prenatal counseling is crucial for managing the situation and identifying specialist areas. Gestational diabetes can also help prevent and lower NTD incidence [32].

As exhibited in most of our case reports, women who are in the reproductive age bracket and are at risk of NTDs may be uneducated or economically disadvantaged, have a family history of NTDs, have no folic acid supplements in pregnancy, have weakness or fatigue, suffer from a type of dyspnea associated with anemia, are diabetic, and have been prescribed antiepileptic drugs [27] Moreover, research demonstrates a significant reduction of fetal neural tube disorders among mothers taking periconceptional dietary supplementation with folic acid, where all mothers should take at least 0.4–0.8 mg of folic acid every day [33]. Mothers are advised to prevent recurrence of NTD if they have experienced it in the past or have delivered a child with defective NTD, by observing daily ingestion of 4 mg folic acid commencing a month prior to conception and 3 months postconception. It is estimated in research studies that the duration it takes for the red cells to return to their normal levels is at least 20 weeks [34].

On another level, it is important to note that the occurrence of an occipital encephalocele in a diamniotic dichorionic twin pregnancy is an extremely rare phenomenon. Our third case report sheds light on the specific occurrence and also underlines the role of advanced imaging techniques, such as 3D ultrasonography, in prenatal diagnosis. Nonetheless, despite the favorable Apgar scores, the affected neonate′s subsequent demise due to sepsis reflects the limitations in neonatal intensive care units (NICUs) in Lebanon [35, 36], particularly when it comes to the management of complex congenital anomalies. However, the presence of a healthy cotwin proves that managing such presentations is case specific and that individualized care plans should be adopted in multifetal pregnancies.

### 6.1. Patient Education and Knowledge

Because of nutritional unawareness, NTDs are not uncommon especially among the members of the community who are not engaged in health promotion activities. Possible prevention techniques demand the supplementation of folic acid as well as antiseizure medications for women of childbearing age. At 4 months after conception, it is advised for blood tests and ultrasound scans to be performed to pregnant mothers at high clinical risk for congenital anomalies and NTDs. Any guidelines for surgical correction of the anomaly should be incorporated in a nationwide campaign, together with genetic counseling. The healthcare system response to NTDs should be holistic and must involve an interprofessional team that includes genetic analysts as well as specialists of cardiology, nephrology, neurology, and rheumatology [1, 25, 37].

## 7. Conclusion

Neurological disorders (NTDs) are a significant cause of multineonatal mortality and morbidity, with spina bifida and anencephaly being the most common. Folic acid deficiency is a risk factor for NTDs, suggesting proper supplementation for pregnant women. This is especially important in the Lebanese context which lacks adequate public health awareness measures and prenatal health access for vulnerable populations. Pregnant women should take 400 *μ*g of folic acid daily 4 weeks before conception, continuing until the 12th week. Prenatal and postnatal management should include ultrasound, clinical signs, and tertiary centers with trained teams.

## Author Contributions

Dr. Kariman Ghazal and Mr. Christopher Massaad drafted the manuscript, collected data, and ensured the accuracy of the information. Dr. Nidal Hammoud collected data. Mr. Christopher Massaad was responsible of writing, reviewing, and editing the article, in addition to the visualization and formal analysis. Dr. Charlotte El Hajjar provided supervision. Dr Georges Yared edited the final version of the manuscript while providing supervision.

## Funding

No funding was received for this manuscript.

## Ethics Statement

This study has received ethics approval from the local institutional review board. Due to the ongoing crisis and economic difficulties in Lebanon, formal documentation and approval numbers are currently unavailable. Dr. Georges Yared, who is responsible for the board′s approvals, has overseen and endorsed this process.

## Consent

Written informed consent was obtained retrospectively from the parents/legal guardians of all four neonates in this case series.

## Conflicts of Interest

The authors declare no conflicts of interest.

## Data Availability

The data supporting the findings of this study are available upon request from the corresponding author.
